# Risk Factors for Focal Choroidal Excavation Concurrent with Chorioretinal Disease: Evaluated by Spectral-Domain OCT

**DOI:** 10.1016/j.xops.2024.100554

**Published:** 2024-05-22

**Authors:** Yiwen Ou, Minghui Qiu, Mengyuan Li, Yajun Mi, Dezheng Wu, Shibo Tang, Weiwei Dai, Jacey Hongjie Ma

**Affiliations:** 1 Aier Academy of Ophthalmology, Central South University, Changsha, Hunan, China; 2 Department of Retinal and Vitreous Diseases, Foshan Aier Eye Hospital, Foshan, Guangdong, China; 3 Department of Retinal and Vitreous Diseases, Guangzhou Aier Eye Hospital, Guangzhou, Guangdong, China; 4 Department of Retinal and Vitreous Diseases, Changsha Aier Eye Hospital, Changsha, Hunan, China

**Keywords:** FCE, Chorioretinal disorders, Multimodal imaging, Risk factor, SD-OCT

## Abstract

**Purpose:**

To investigate the risk factors for patients with focal choroidal excavation (FCE) and their correlation with chorioretinal diseases.

**Design:**

Retrospective cross-sectional study.

**Subjects:**

Patients with FCE were enrolled, while healthy subjects were recruited for the control group.

**Methods:**

The study collected demographic information, clinical features, and multimodal images. Parameters of FCE identified using spectral-domain OCT (SD-OCT) were manually measured using built-in software and subsequently analyzed statistically.

**Main Outcome Measures:**

Subfoveal choroidal thickness (SFCT), subexcavation choroidal thickness (SECT), and the greatest depth and width of each excavation were manually measured using built-in calipers in OCT software.

**Results:**

Twenty-one patients (13/8, male/female) with FCE were included in this study. The average age was 45.2 years, and their best-corrected visual acuity (BCVA) was 0.4 logarithm of the minimum angle of resolution (Snellen equivalent, 20/50). Focal choroidal excavation was present in 28 eyes of 21 patients, including isolated FCE (12 eyes) and complicated FCE (16 eyes) with choroidal neovascularization (sCNV), central serous chorioretinopathy, and other conditions. Patients with complicated FCE were significantly older than those isolated FCE (*P* = 0.015). The SFCT of the healthy subjects was significantly less than that of the fellow eyes of the patients with FCE (*P* < 0.01), as was that of the eyes with isolated FCE (*P* < 0.001) and complicated FCE (*P* < 0.001). The width of excavation was wider in eyes with complicated FCE than in those with isolated FCE (*P* = 0.001). Hypertransmission defect (HD) was found beneath 15 excavations and was more prevalent in the complicated FCE group than the isolated FCE group (*P* = 0.023).

**Conclusions:**

Focal choroidal excavation appears to be closely related to chorioretinal disorders, and the width of the excavation is a significant indicator for evaluating the risk of chorioretinal diseases.

**Financial Disclosure(s):**

Proprietary or commercial disclosure may be found in the Footnotes and Disclosures at the end of this article.

Focal choroidal excavation (FCE) is characterized as a clinical condition in which the outer retina is indented into the choroid, with no accompanying scleral changes (such as the absence of staphyloma or scleral ectasia) in the foveal or parafoveal regions.^1^ It is most frequently observed in young and middle-aged patients and has no significant effect on sex.^2^ Patients with FCE typically exhibit unilateral symptoms, often with or without mild metamorphopsia, and more than 90% of these patients are Asian.^3^ Because the affected eyes usually maintain good visual acuity and lesions exhibit minimal changes over time, concavity in the choroid was once thought to be merely a congenital choroidal malformation.^4^

As previously reported, FCE may be concurrent with sight-threatening chorioretinal disorders, including choroidal neovascularization (CNV), central serous chorioretinopathy (CSC), and polypoidal choroidal vasculopathy.^5^ Recently, FCE was considered to contribute to the development of choroidal disease. However, the mutual cause and effect have still been obscured. However, whether chorioretinal diseases can lead to FCE or occur as a complication of FCE is still unknown.

To decipher the risk factors for FCE and chorioretinal diseases, we investigated patients' clinical characteristics and imaging data of the excavation. The present study provided detailed data on patients with FCE, including demographic characteristics and fundus findings obtained using spectral-domain OCT (SD-OCT) and multimodal imaging.

## Methods

This study received approval from the Ethics Committee of Guangzhou Aier Eye Hospital (GZAIER2020IRB10) and was conducted in accordance with the tenets of the Declaration of Helsinki. Written informed consent was obtained from each subject before any study procedures or examinations were performed.

Patients diagnosed with FCE who visited Guangzhou Aier Eye Hospital between August 2016 and August 2022 were enrolled in the study. The inclusion criterion for this study was the presence of FCE, as observed via SD-OCT. Focal choroidal excavation was defined as the outward deformation of the photoreceptor tips and the retinal pigment epithelium (RPE)/Bruch's membrane complex, identified on an SD-OCT scan, in the absence of posterior staphyloma or scleral ectasia. Choroidal neovascularization was diagnosed based on symptoms including visual impairment or metamorphopsia, subretinal fluid or cystoid spaces, subretinal hyperreflective material, and disruption in the RPE layer on OCT or angiographic signs of leakage. The diagnosis of CSC relies on medical history, detection of serous retinal detachment in the macular region confirmed by fundoscopic examination, and OCT, along with fluorescein leakage. The exclusion criterion included any previous history of posterior segment trauma or tumor.

In accordance with the extent of outer retinal structure destruction during excavation, we categorized this destruction into mild and severe groups (Fig S1, available at www.ophthalmologyscience.org). The mild group was defined by outer retinal structures, including the external limiting membrane, ellipsoid zone, interdigitation zone, and RPE/Bruch's complex, which remained continuous or exhibited slight disturbances such as thinning or attenuation over the excavation. In contrast, the severe group exhibited outer retinal structures that were partially or completely disrupted. Hypertransmission defects (HDs), defined by the Classification of Atrophy Meetings,^6^^,^^7^ are characterized as unusually highly reflective tissue connecting the bottom of the excavation to the outer choroidal boundary (Fig S2, available at www.ophthalmologyscience.org).

All patients' clinical data, encompassing demographic information, best-corrected visual acuity (BCVA), intraocular pressure, and slit-lamp biomicroscopy, were recorded. Multimodal imaging data were also gathered, including color fundus photography (Visucam 524; Zeiss), Optos cSLO (OPTOS PLC Daytona P200T), SD-OCT (Spectralis; Heidelberg Engineering), OCT angiography (Cirrus 5000 Angioplex; Zeiss Meditec), fundus autofluorescence (FAF), fluorescein fundus angiography (FFA), and indocyanine green angiography (ICGA) (Spectralis HRA + OCT; Heidelberg Engineering). Patients with FCE with chorioretinal diseases were diagnosed by experienced senior consultant doctors via clinical examination, SD-OCT, and angiography.

**Figure 3:**
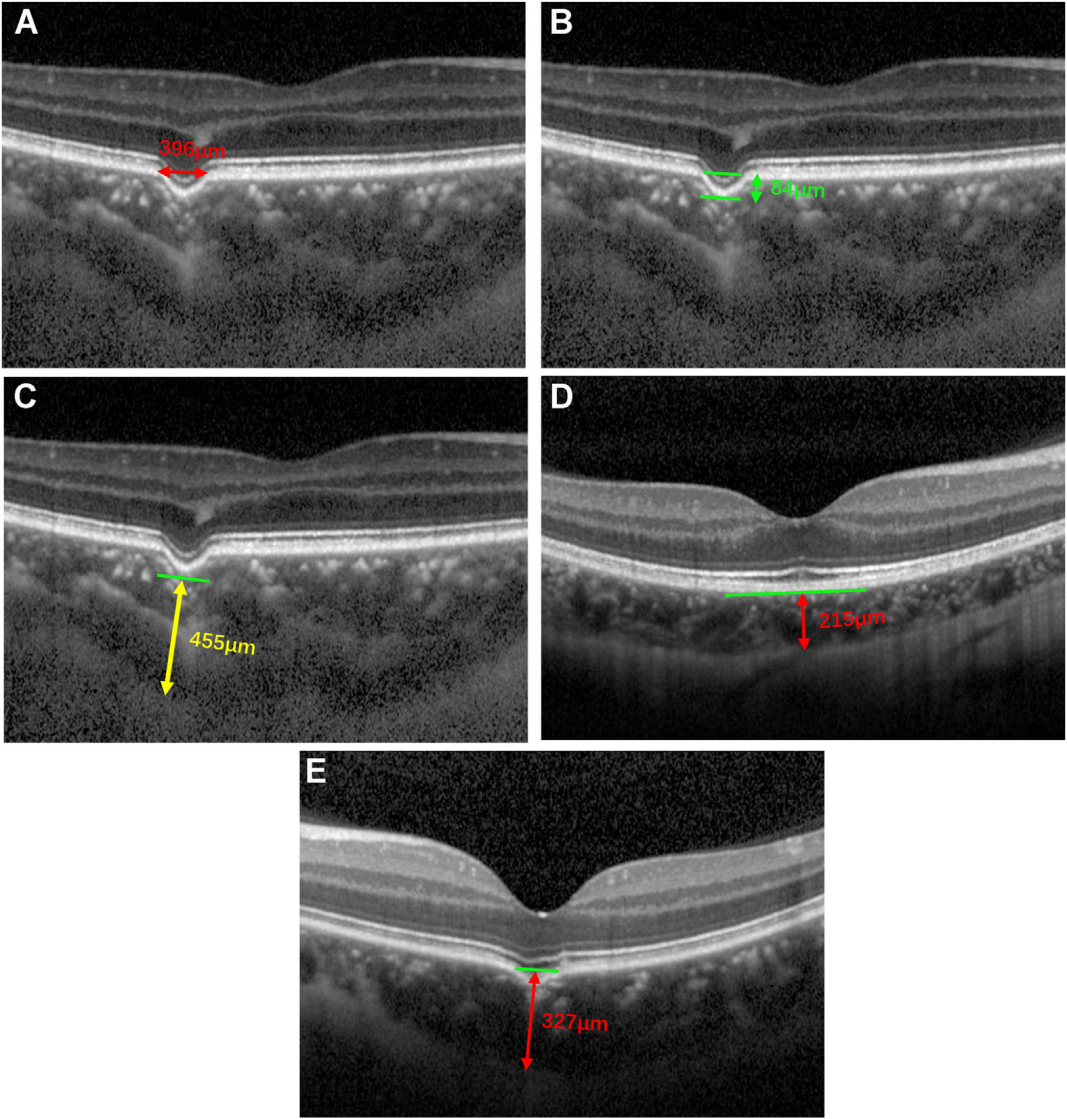
Measurement of focal choroidal excavation parameters. **A,** Measurement of the greatest linear diameter (red double-headed arrow) at the excavation. **B,** Measurement of depth (green double-headed arrow) at the excavation. **C,** Measurement of the choroidal thickness at the base of the excavation. **D,** Measurement of subfoveal choroidal thickness in the fellow eye of patients with focal choroidal excavation (red). **E,** Measurement of subfoveal choroidal thickness in eyes excavated in the macular region.

Spectral-domain OCT raster scans were performed on each eye centered at the fovea using 2 scan protocols: a conventional protocol for detecting FCE and an enhanced depth imaging protocol for observing the choroid. Subfoveal choroidal thickness (SFCT), subexcavation choroidal thickness, and the greatest depth and width of each excavation were manually measured using built-in calipers in OCT software. Specifically, the greatest width of an excavation was measured as the distance between uninvolved adjacent RPE layers on both sides of the FCE (Fig 3A). The greatest depth of the excavation was measured from the line indicating the width to the outer border of the lowest tip of the excavated RPE layer (Fig 3B). The subexcavation choroidal thickness measurement was determined as the distance from the outer border of the lowest point of the excavated RPE layer to the inner scleral surface (Fig 3C). Subfoveal choroidal thickness was measured as the distance from the Bruch's membrane to the inner scleral surface (Fig 3D). If the excavation was located beneath the foveal center, the SFCT was measured from the projected RPE/Bruch's membrane complex (the 2 points where the RPE/Bruch's membrane complex bulges outward) to the inner scleral surface (Fig 3E).

**Figure 4:**
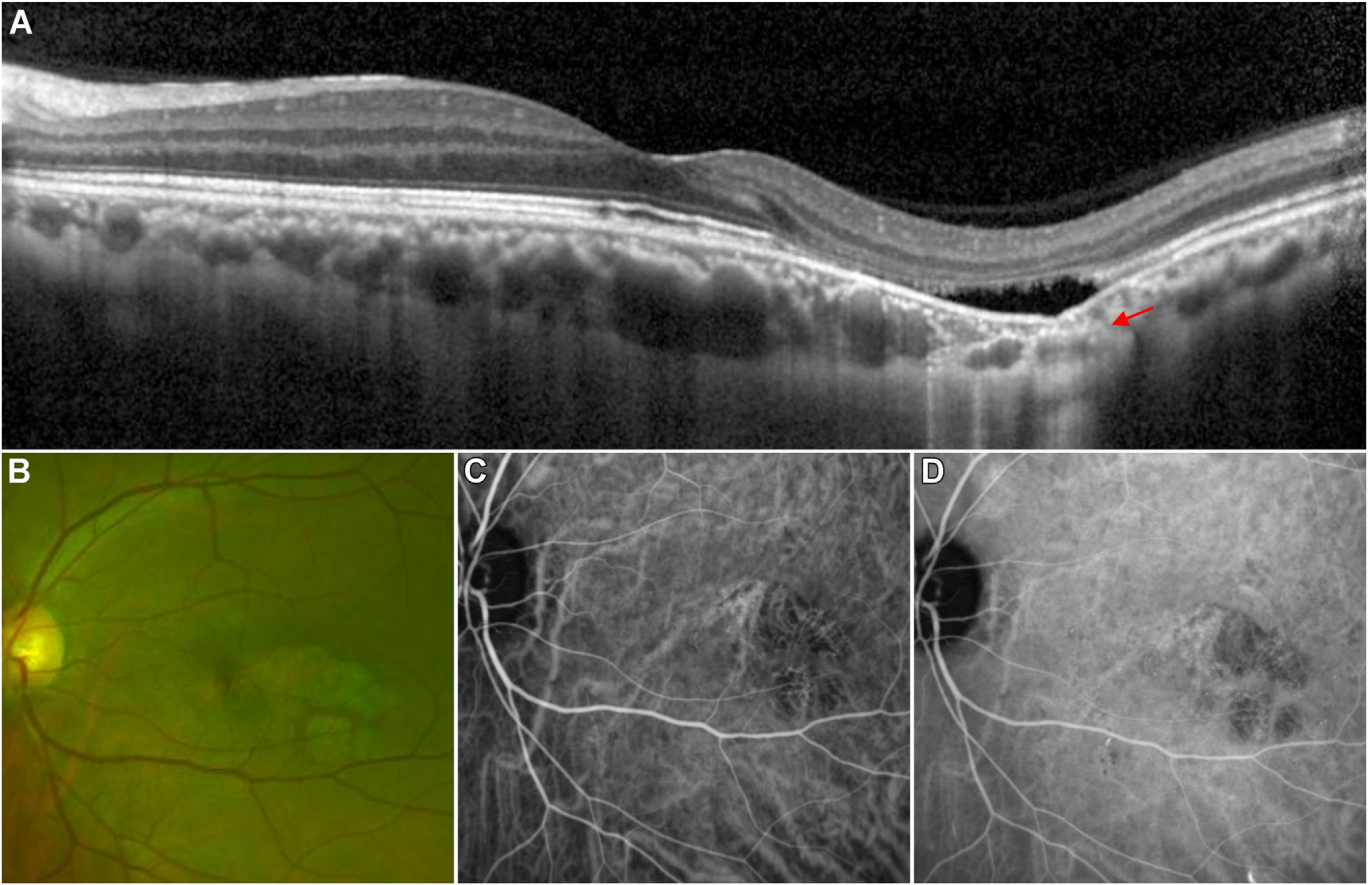
A 51-year-old male with complicated focal choroidal excavation in the left eye with blurred vision. **A,** Spectral-domain OCT imaging of the left eye revealed a large extrafoveal choroidal excavation, accompanied by a shallow neurosensory retinal detachment overlying the excavation. The choroid appeared compressed and thinned beneath the excavation, and hyperreflective tissue under the focal choroidal excavation formed a bridge between the bottom of the excavation and the outer choroidal boundary (red arrow). **B,** Optomap scanning laser ophthalmoscopy revealed extrafoveal subretinal yellowish-white plaque lesions. Indocyanine green angiography ([**C**] early phase; [**D**] late phase) demonstrated hypofluorescence at the excavation site and the surrounding area, with dilated vessels.

**Figure 5:**
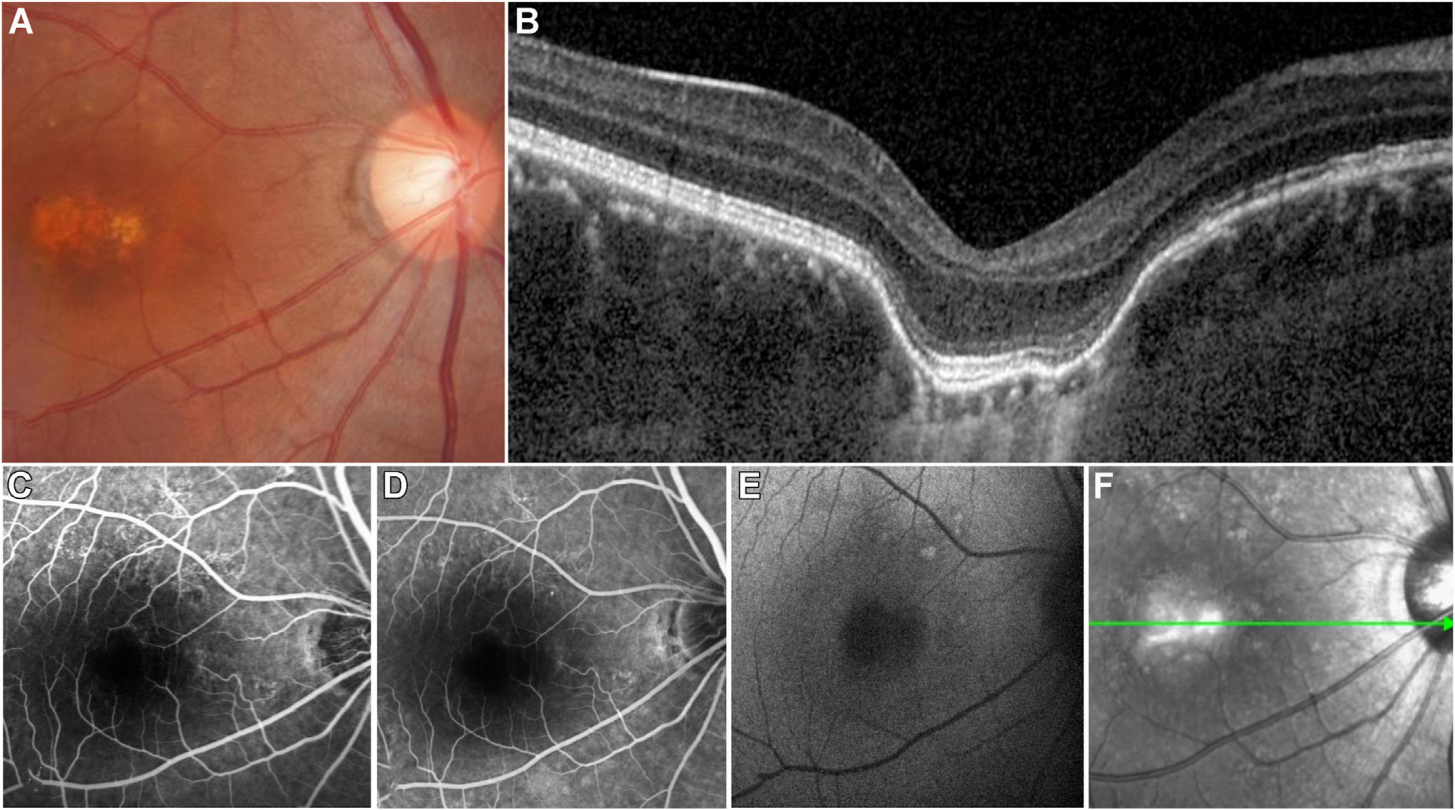
A 31-year-old male with isolated focal choroidal excavation complained of metamorphopsia in the right eye. **A,** Color fundus photography revealed a yellowish oval lesion at the fovea. **B,** OCT imaging revealed the external limiting membrane, ellipsoid zone, and retinal pigment epithelium conforming to the contour of the choroidal excavation; slight disturbances were observed in the outer retina. Additionally, compared with that in the surrounding areas, the outer nuclear layer overlying the choroidal excavation was thicker. **C** and **D,** Fluorescein fundus angiography revealed an expanded hypofluorescent area corresponding to focal choroidal excavation beneath the fovea and presented irregular hyperfluorescence above the excavation without leakage in the later phase. **E,** Fundus autofluorescence imaging showed central hypoautofluorescence. **F,** Infrared reflectance imaging revealed central hyperreflective areas surrounded by irregular hyporeflective signals.

**Figure 6:**
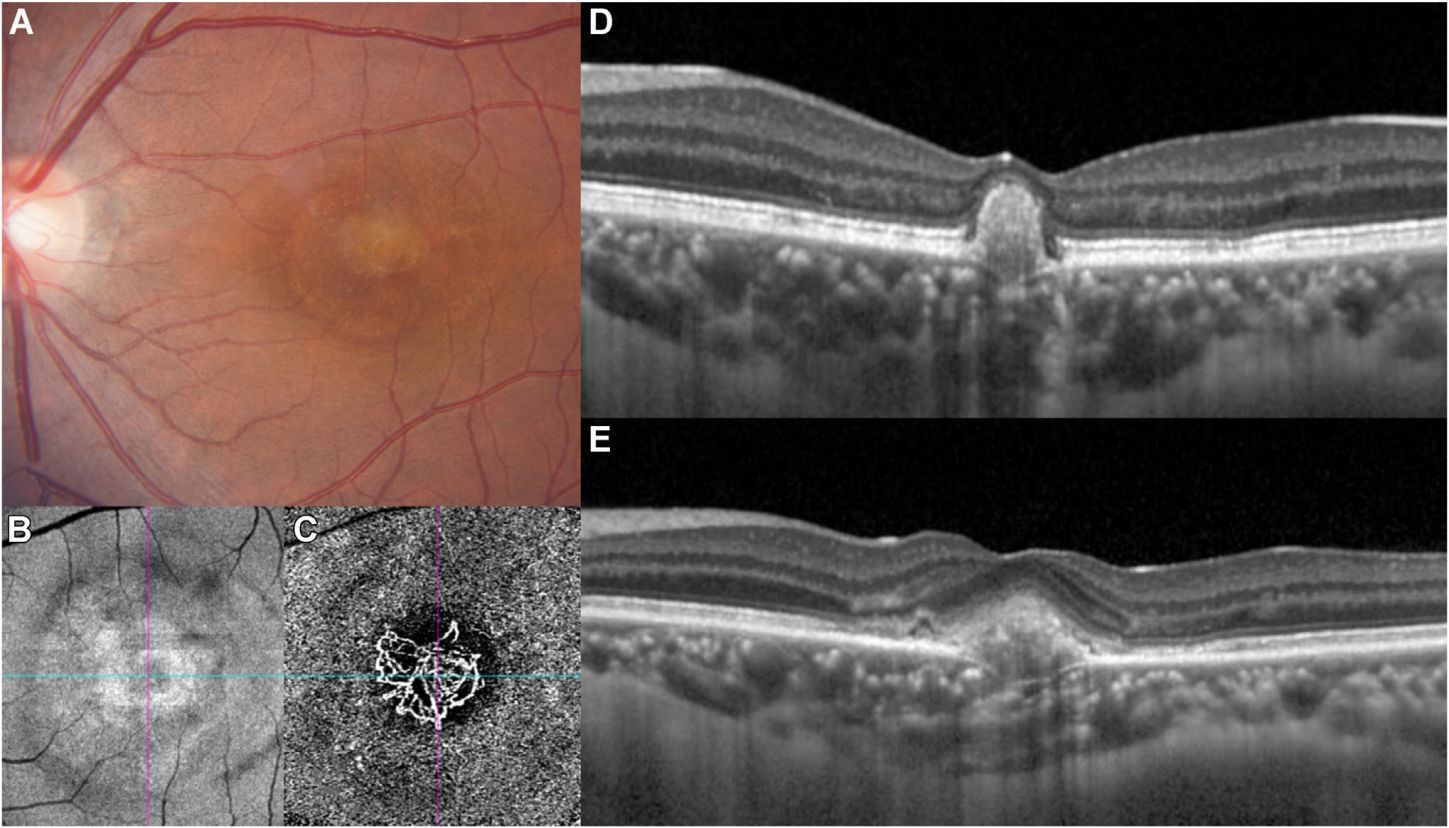
A 33-year-old female with complicated focal choroidal excavation occurring concurrently with choroidal neovascularization. **A,** Color fundus photography revealed a grayish lesion at the fovea. **B** and **C,** OCTA image showing a well-defined choroidal neovascularization beneath the lesion at the fovea in en face structural (**B**) and angiographic (**C**) images. **D,** Spectral-domain OCT revealed hyperreflective foci disrupting the retinal pigment epithelium/Bruch's membrane complex extending to the external limiting membrane. **E,** After 3 years of follow-up, there was an increase in both the depth and width of the excavation.

The BCVA was measured using a Snellen chart and subsequently converted into a logarithm of the minimum angle of resolution (logMAR) for statistical analysis. Quantitative data are presented as the means ± standard deviations. The Mann‒Whitney *U* test was used to compare the means of numerical variables if they were normally distributed, otherwise, the Wilcoxon signed-rank test was utilized. Fisher exact test was used to compare the distributions of categorical variables. A *P* value of 0.05 was considered to indicate statistical significance.

## Results

### Demographics and Clinical Features

The demographic data of the patients with FCE are summarized in Table 1. Twenty-eight eyes from 21 patients were enrolled in the study, 13 of whom were men. All enrolled subjects were Chinese. Focal choroidal excavation was bilateral in 7 patients and unilateral in 14 patients, the ratio of affected left to right eyes was 5/9. The mean age of the patients was 45.2 years (ranging from 27 to 70 years), with an average BCVA of 0.4 logMAR (Snellen acuity of 20/50).

**Table 1:** Demography and Clinical Information of Patients with Focal Choroid Excavation

Patient ID	Age/Gender	BCVA (logMar)	Laterality	Number of Excavation	Primary Diagnosis	Fellow Eye Diagnosis
1	28/M	0	L	1	Isolated	Normal
2	34/M	0.3	R	3	Isolated	NA
		−0.2	L	1	Isolated	NA
3	34/F	0	R	3	Isolated	Normal
4	43/F	0	R	1	Isolated	NA
		−0.2	L	1	Isolated	NA
5	51/M	0	L	1	CSC	PPE
6	36/M	0.6	R	1	CNV	NA
		0.6	L	1	PIC	NA
7	41/F	1	R	1	CNV	NA
		0	L	1	Isolated	NA
8	56/F	0.2	R	2	CNV	NA
		0	L	1	Isolated	NA
9	34/F	0.7	L	1	CNV	Normal
10	42/F	0	R	1	CSC	Normal
11	48/M	1	R	1	CSC	NA
		0.5	L	1	CSC-CNV	NA
12	51/M	0.1	R	1	Isolated	Normal
13	48/M	0.5	R	1	MH	MEM
14	40/M	1	R	1	RD	Normal
15	70/M	1	R	1	MEM	Normal
16	27/F	0	R	1	Isolated	Normal
17	69/M	0.7	L	1	CSC	PPE
18	69/M	0.4	L	1	CNV	Normal
19	43/M	1	R	1	CNV	Normal
20	38/M	0	R	1	Isolated	Normal
21	48/F	0.1	R	1	Isolated	NA
		0.7	L	2	CNV	NA

BCVA = best-corrected visual acuity; CNV = choroidal neovascularization; CSC = central serous chorioretinopathy; F = female; ID = identity document; L = left; logMAR = logarithm of the minimum angle of resolution; M = male; MEM = macular epiretinal membrane; MH = macular hole; NA = not available; PIC = punctate inner choroidal; PPE = pachychoroid pigment epitheliopathy; R = right; RD = retinal detachment.

Twenty-one healthy individuals, matched for age and sex (35 eyes), served as controls for the study. No statistically significant difference was observed in the mean age between FCE patients and healthy controls (43.0 ± 12.4 years vs. 42 ± 14.4 years, *P* = 0.457).

### Types of FCE on SD-OCT

Based on the morphology observed in SD-OCT images, FCE can be classified into 2 types: nonconforming (Fig 4A), which features a separation between the photoreceptor tips and the RPE, and conforming (Fig 5B), which exhibits no such separation. The study identified 26 conforming lesions and 8 nonconforming lesions.

FCE can be classified as foveal (<200 μm) or extrafoveal (≥200 μm) depending on the distance between its posterior border and the fovea. In our FCE cases, 30 were located subfoveally, and 4 were extrafoveally located.

### Complicated FCE vs. Isolated FCE

Thirty-four FCE lesions were identified in 28 eyes of 21 patients using SD-OCT (Table 2). Based on the presence of chorioretinal disease, the affected eyes were divided into 2 groups: isolated FCE (12 eyes) and complicated FCE (16 eyes). Among the 16 eyes with complicated FCE, 7 had CNV, 3 had central CSC, 3 had choroiditis, one had a macular hole, one had a macular epiretinal membrane, and one had retinal detachment. Twelve fellow eyes were from the opposite eye of unilateral patients with FCE without any excavation. Patients with complicated FCE were significantly older than were those with isolated FCE (47.4 ± 11.9 years vs. 34 ± 8.0 years, *P* = 0.015). The BCVA in patients with complicated FCE was worse than that in patients with isolated FCE (0.62 ± 0.34 vs. 0.01 ± 0.13 in logMAR, 20/5 ± 20/9 vs. 20/16 ± 20/15 in Snellen, *P* < 0.001) (Table S3, available at www.ophthalmologyscience.org).

**Table 2:** Morphological and Tomographic Characteristics of FCE

FCE ID	Group	Type of FCE	Parameters		Choroid Thickness			Outer Retinal Structure and Choriod			
			Width	Depth	SFCT	CTUE	SFCT in Fellow Eye	HD	EZ	IZ	RPE
FCE1-1os	I	Conforming	560	61	327	235	244	−	Intact	Disturbed	Disturbed
FCE2-1od	I	Nonconforming	1039	269	343	55	NA	+	Disrupted	Disrupted	Disturbed
FCE2-2od	I	Conforming	912	302	343	36	NA	+	Disrupted	Disrupted	Disturbed
FCE2-3od	I	Conforming	199	54	343	305	NA	−	Intact	Intact	Intact
FCE2-1os	I	Conforming	164	43	363	313	NA	−	Intact	Intact	Disturbed
FCE3-1od	I	Conforming	396	64	487	152	469	−	Intact	Disrupted	Disturbed
FCE3-2od	I	Conforming	199	58	487	331	469	−	Disturbed	Disturbed	Disturbed
FCE3-3od	I	Conforming	292	39	487	318	469	−	Disturbed	Disturbed	Disturbed
FCE4-1od	I	Conforming	207	58	242	208	NA	−	Disrupted	Disrupted	Disturbed
FCE4-1os	I	Conforming	402	114	169	135	NA	−	Disturbed	Disturbed	Intact
FCE5-1os	C	Nonconforming	2288	186	284	100	327	+	Disrupted	Disrupted	Disturbed
FCE6-1od	C	Conforming	579	48	358	279	NA	+	Intact	Disturbed	Disrupted
FCE6-1os	C	Conforming	915	49	308	162	NA	+	Disrupted	Disrupted	Disturbed
FCE7-1od	C	Conforming	1030	55	306	161	NA	+	Disrupted	Disrupted	Disturbed
FCE7-1os	I	Conforming	365	49	289	300	NA	−	Intact	Intact	Intact
FCE8-1od	C	Nonconforming	763	111	369	253	NA	+	Disrupted	Disrupted	Disturbed
FCE8-2od	C	Conforming	618	81	369	279	NA	+	Disrupted	Disrupted	Disturbed
FCE8-1os	I	Conforming	235	39	326	271	NA	−	Intact	Disturbed	Disturbed
FCE9-1os	C	Conforming	466	73	326	284	363	−	Disrupted	Disrupted	Disrupted
FCE10-1od	C	Nonconforming	260	75	162	110	190	+	Disrupted	Disrupted	Disrupted
FCE11-1od	C	Conforming	3234	202	432	222	NA	−	Disrupted	Disrupted	Disturbed
FCE11-1os	C	Conforming	1878	136	423	272	NA	−	Disrupted	Disrupted	Disrupted
FCE12-1od	I	Conforming	793	104	77	28	148	+	Intact	Intact	Disturbed
FCE13-1od	C	Nonconforming	680	183	177	72	106	+	Disrupted	Disrupted	Disturbed
FCE14-1od	C	Conforming	749	56	128	81	227	−	Disrupted	Disrupted	Disturbed
FCE15-1od	C	Conforming	346	57	204	201	NA	−	Disturbed	Disturbed	Disturbed
FCE16-1od	I	Conforming	456	75	333	288	288	−	Disrupted	Disrupted	Disturbed
FCE17-1os	C	Nonconforming	577	80	279	194	159	−	Disturbed	Disturbed	Disturbed
FCE18-1os	C	Nonconforming	496	50	149	80	NA	+	Disturbed	Disturbed	Disturbed
FCE19-1od	C	Conforming	553	112	400	132	356	+	Disturbed	Disturbed	Disturbed
FCE20-1od	I	Nonconforming	471	107	540	274	434	−	Disturbed	Disrupted	Disturbed
FCE21-1od	I	Conforming	358	59	505	384	NA	−	Intact	Intact	Intact
FCE21-1os	C	Conforming	3003	185	394	243	NA	+	Disrupted	Disrupted	Disrupted
FCE21-2os	C	Conforming	1539	140	394	278	NA	+	Disrupted	Disrupted	Disrupted

C = complicated; CTUE = choroidal thickness under the excavation; EZ = ellipsoid zone; FCE = focal choroid excavation; HD = Hypertransmission defect; I = isolated; ID = identity document; IZ = interdigitation zone; NA = not available; RPE = retinal pigment epithelium; SFCT = subfoveal choroidal thickness.

The SFCT in the isolated FCE group (333.4 ± 134.8 μm) and the fellow eye in the isolated FCE group (316.6 ± 133.7 μm) were thicker than those in the control group (213.4 ± 45 μm, *P* < 0.001). However, there was no significant difference between the isolated FCE group and its fellow eye (*P* = 0.81) (Table S4, available at www.ophthalmologyscience.org). The SFCT in the complicated FCE group (293.7 ± 102 μm) and the fellow eye in the complicated FCE group (246.9 ± 102.5 μm) were thicker than those in the control group (213.4 ± 45 μm, *P* < 0.001). However, there was no significant difference between the complicated FCE group and the fellow eye (*P* = 0.32) (Table S5, available at www.ophthalmologyscience.org). Furthermore, there was no significant difference in the SFCT between the isolated FCE group and the complicated FCE group (*P* = 0.382).

The average minimum thickness at the deepest point of FCE was not significantly different between the complicated FCE group and the isolated FCE group (227.1 ± 112.7 μm vs. 189.1 ± 78.4 μm, *P* = 0.147). The width of the FCE was greater in the complicated FCE group than in the isolated FCE group (1109.7 ± 906.0 μm vs. 440.4 ± 264.2 μm, *P* < 0.001), while the depth of the FCE was similar between the 2 groups (104.4 ± 54.5 μm vs. 95.4 ± 78.1 μm, *P* = 0.702) (Table S6, available at www.ophthalmologyscience.org).

### Mild vs. Severe Disruption of the Outer Retinal Structure in Patients with FCE

Among the 28 eyes with 34 excavations, 17 had mild destruction, and 17 had severe destruction of the retinal structure. The structures that are typically affected during choroidal excavation include the RPE/Bruch's band, the ellipsoid zone of the photoreceptors, and the external limiting membrane.

Eyes with HD in the outer retina exhibited more severe damage than did those without HDs (*P* < 0.001). Hypertransmission defect was more commonly observed in patients with complicated FCE than in those with isolated FCE (*P* = 0.023) (Table S7, available at www.ophthalmologyscience.org). The choroidal thickness beneath excavations was thinner in the HD group than in the non-HD group (*P* = 0.003). Compared with those in FCE without HD, the excavations in FCE with HD were deeper (*P* = 0.042). However, there were no significant differences in the SFCT or width between the 2 groups (*P* = 0.324, *P* = 0.705) (Table S8, available at www.ophthalmologyscience.org).

### Multimodal Imaging and Clinical Course of FCE

According to the color fundus photographs, all eyes with FCEs exhibited changes in the corresponding area, although the lesions varied based on pigmentation alterations and macular pathology. The lesions presented as pigment disturbances (n = 14), subretinal yellowish-white plaques (n = 6) (Fig 5A), unremarkable changes (n = 4), serous sensory retinal detachments (n = 2), or grayish-white plaques (n = 2) (Fig 6A). On FAF images, 4 FCE lesions displayed varying degrees of hyperfluorescence and hypofluorescence around the excavation. Fluorescein fundus angiography was performed for 4 patients, 3 eyes with concurrent CSC and choroiditis presented leakage of fluorescein dye at the excavation site. While in 1 eye with idiopathic FCE, under the fovea, FFA exhibited an enlarged hypofluorescence area corresponding to the lesion, and punctate hyperfluorescence was shown around the FCE but no leakage in the late phase. In 4 eyes examined with ICGA, choroidal hyperpermeability (Fig 4C and D), along with punctate and schistose hypofluorescent spots, was observed.

The mean follow-up period was 19.8 months (1–42 months). Most FCE lesions showed no enlargement or progression during the follow-up. However, patient 7 had concurrent FCE with CNV at baseline. After 5 anti-VEGF treatments for 3 years, the excavation was enlarged, and the choroid thickness was thinner than initial visit (Fig 6D and E).

## Discussion

With advancements in multimodal imaging, FCE has been found to be present in various chorioretinal diseases, including pachychoroid diseases such as CNV, CSC, age-related macular degeneration and inflammatory diseases such as multiple evanescent white dot syndrome, multifocal choroiditis, and Vogt–Koyanagi–Harada disease.^8^, ^9^, ^10^ However, FCE is identifiable only as a distinct entity through OCT imaging. Currently, there is no consensus on the terminology used to distinguish FCE with and without chorioretinal diseases. Given the unclear cause-and-effect relationship of FCE, we termed excavations without chorioretinal diseases "isolated FCE" and excavations with such diseases "complicated FCE," distinguishing them from terms such as "idiopathic," "primary," "congenital," "acquired," and "secondary." Compared with those in previous studies, the terms "isolated FCE" and "complicated FCE" more accurately depict the conditions of excavations. This pilot study utilized SD-OCT in conjunction with fundus color photography, FAF, FFA, and ICGA and qualitatively and quantitatively analyzed 28 eyes of 21 patients with FCE, aiming to elucidate the risk factors for FCE associated with chorioretinal diseases and the potential mechanisms of excavation formation.

Interestingly, 82% of the excavation was observed under the fovea. The high oxygen demands of the retina and the relatively hypoxic-ischemic nature of the retina are thought to contribute to chorioretinal diseases.^11^ Circulation was more vulnerable to hypoxia and ischemia in the macular region without retinal blood vessels. This pathology and physiology may explain the high incidence of FCE in the fovea compared with other regions. Our study revealed that complicated FCE presented with a greater width of excavation than isolated FCE. We speculated that the greater width of the excavation, accompanied by more severe ischemia of the underlying RPE/Bruch's membrane complex and subsequent disruption of the outer blood–retinal barrier, increase the likelihood of retinochoroidal disorders in these eyes. This close topographic relationship supports the notion that choroidal excavation plays a role in the development of retinochoroidal disorders. More importantly, the width of the excavation is a crucial risk factor for assessing whether macular disease occurs.

Owing to the limited patient population in this research area, a consensus has not yet been established regarding the variation in SFCT among patients with FCE. Chung et al argued that the occurrence of FCE is associated with choroidal thickening, a finding that aligns with our observations.^12^ However, Rajabian et al’s retrospective study indicated that SFCT did not significantly differ from that of the control group.^13^ According to our data, the SFCT in both affected and fellow eyes was significantly greater than that in healthy eyes. Additionally, 2 of the fellow eyes presented with pachychoroid pigment epitheliopathy, an important condition to monitor.

Recently, FCE has been recognized as part of the pachychoroid spectrum of diseases.^14^, ^15^, ^16^, ^17^ Our case series demonstrated that 57.1% of the FCE-involved eyes had accompanying chorioretinal diseases, with 62.5% having pachychoroid diseases; notably, CNV constituted 70% of these eyes. The prevalence of CNV among the chorioretinal diseases in our study was in line with that in previous reports.^18^^,^^19^ In our study, all CNV cases occurred within the excavation, leading us to infer that focal disruptions and ischemia of the RPE/Bruch's complex might predispose individuals to CNV development. The distortion and weakening of the RPE/Bruch's membrane complex around the excavation facilitated neovascular growth into the area. In 1 patient (patient 7) with complicated CNV, the width and depth of the excavation increased following 5 anti-VEGF treatments over 3 years. We hypothesize that chorioretinal diseases such as CNV may contribute to the development of fractures, and the traction exerted by neovascular forces on the weakened RPE/Bruch's membrane toward the choroid is a potential factor in FCE lesion development. However, the exact cause-and-effect relationship between FCE and CNV remains to be determined.

Investigators found that HD on OCT beneath the excavation ranged from 19.4% to 55.6%,^20^^,^^21^ compared to the 44.1% observed in our study. Our novel findings indicate that HD is more prevalent in patients with a severely damaged outer retina and often co-occurs with retinochoroidal disorders. Additionally, we observed that the choroidal thickness beneath excavations was significantly lower in patients who underwent HD than in those who did not. Choroidal exudation, resulting from inflammation, not only weakens the RPE/Bruch's complex but also leads to the pulling of the choroid stroma and RPE/Bruch's membrane toward the outer border of the choroid, followed by the development of subretinal fibrin.

The limitations of our study include a small sample size due to the low prevalence of this condition, short follow-up period, and retrospective design. It has been reported that FCE is more likely presented in the myopia cases.^6^^,^^12^ However, in the present study, we overlooked the effect of refractive status on the FCE, which should be considered as a limitation of our study. A more extensive longitudinal follow-up study involving a larger number of patients and an extended follow-up period is necessary to comprehensively understand the natural progression, pathophysiology, and clinical effects of FCE.

In conclusion, chorioretinal diseases, especially CNV, are frequently observed in patients with FCE. Complicated FCE is more common in older individuals, and the width of the excavation serves as a significant indicator for evaluating the risk of complicated FCE. Although most FCEs appear to remain stable, the change in the choroid around the excavation indicates that regular follow-up is essential for monitoring FCE eyes.
