# Supplementary material for: Risk Factors for Focal Choroidal Excavation Concurrent with Chorioretinal Disease: Evaluated by Spectral-Domain OCT
Source: Ophthalmol Sci. 2024 May 22;4(6):100554. doi: 10.1016/j.xops.2024.100554 (PMC11324813; doi:10.1016/j.xops.2024.100554)
Supplement: Figure S1 [file mmc1.pdf]

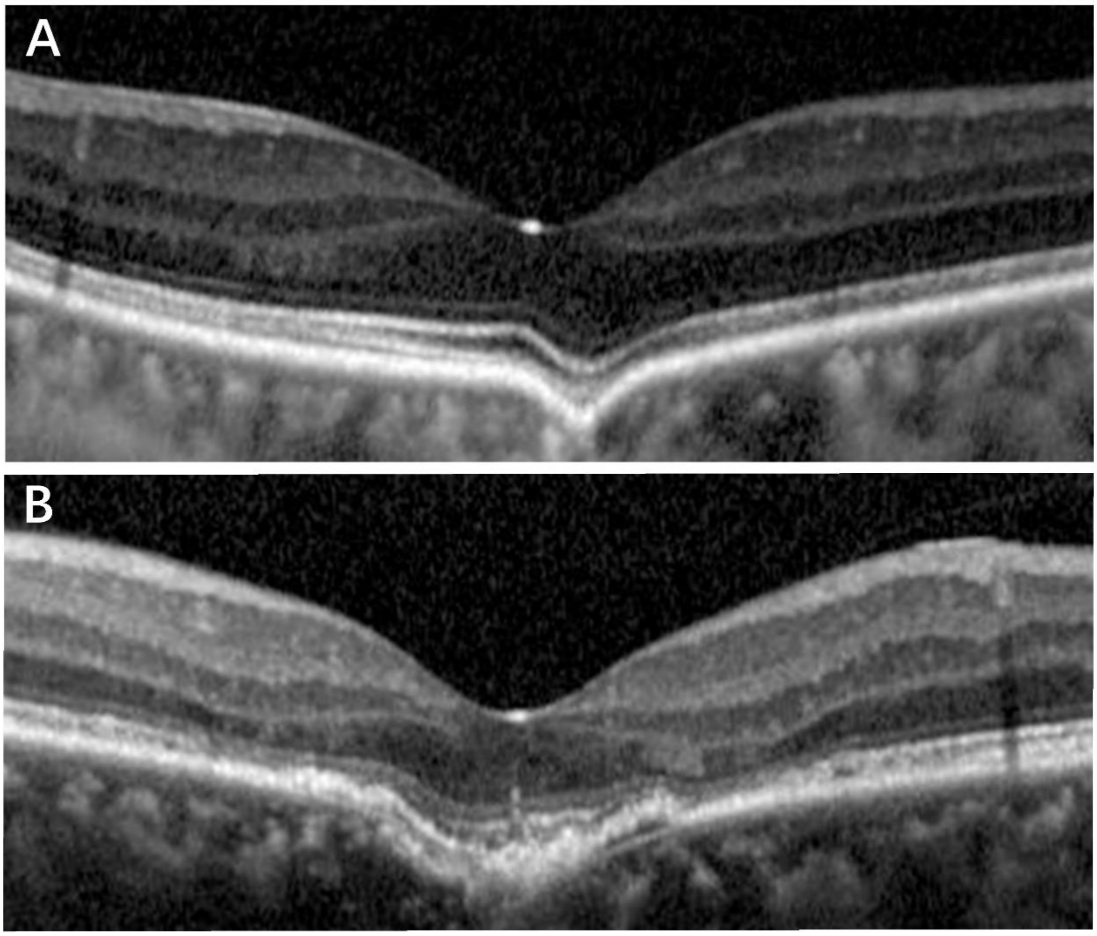

Figure S1. Definition of retinal structure destruction. A, Mild group: Outer retinal structures, including the external limiting membrane (ELM), ellipsoid zone (EZ), interdigitation zone (IZ), and RPE/Bruch's complex, remained intact or showed minor disruptions as thinning or attenuation above the excavation area. B, Severe group: Outer retinal structures disrupted partially or completely.
