# Supplementary material for: Risk Factors for Focal Choroidal Excavation Concurrent with Chorioretinal Disease: Evaluated by Spectral-Domain OCT
Source: Ophthalmol Sci. 2024 May 22;4(6):100554. doi: 10.1016/j.xops.2024.100554 (PMC11324813; doi:10.1016/j.xops.2024.100554)
Supplement: Figure S2 [file mmc2.pdf]

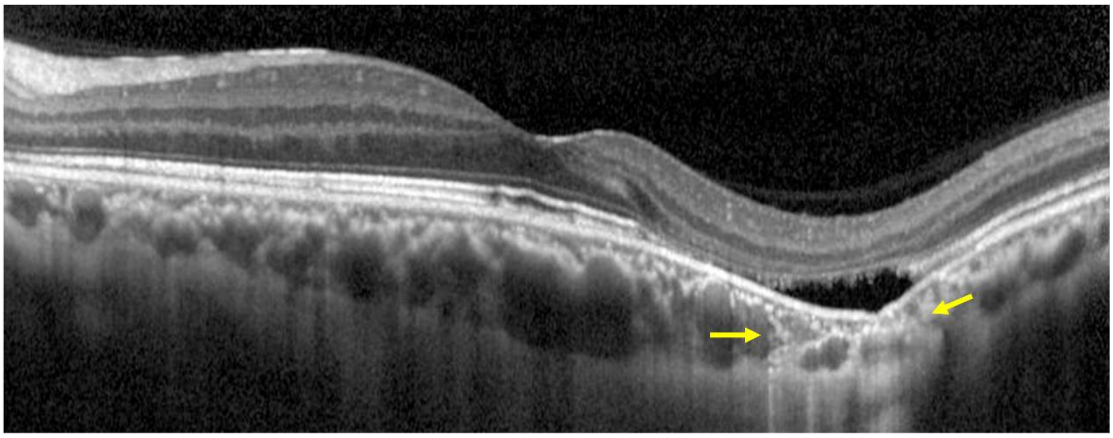

Figure S2. Hypertransmission Defects (HD, between yellow arrows) beneath the excavation are indicated as an unusually high reflectivity in tissue, forming a connection from the bottom of the excavation to the outer choroidal boundary.
