# Supplementary material for: Risk Factors for Focal Choroidal Excavation Concurrent with Chorioretinal Disease: Evaluated by Spectral-Domain OCT
Source: Ophthalmol Sci. 2024 May 22;4(6):100554. doi: 10.1016/j.xops.2024.100554 (PMC11324813; doi:10.1016/j.xops.2024.100554)
Supplement: Table S3 [file mmc3.pdf]

Table S3. Comparison of clinical data between IFCE and CFCE groups

	IFCE	CFCE	Standard value	P value
Age(Y)	34±8	47.4±11.9	z=-2.431	P=0.015
Visual acuity (logMAR)	0.01±0.13	0.62±0.34	z=-3.886	P < 0.001

IFCE=isolated focal choroid excavation; CFCE=complicated focal choroid excavation; Y=year.
