# Supplementary material for: Risk Factors for Focal Choroidal Excavation Concurrent with Chorioretinal Disease: Evaluated by Spectral-Domain OCT
Source: Ophthalmol Sci. 2024 May 22;4(6):100554. doi: 10.1016/j.xops.2024.100554 (PMC11324813; doi:10.1016/j.xops.2024.100554)
Supplement: Table S4 [file mmc4.pdf]

Table S4. Comparison of SFCT in IFCE, fellow eye of IFCE and healthy groups

	SFCT(μm)	P1	P2
IFCE	333.4±134.8	-	-
fellow eye of IFCE	316.6±133.7	0.81	-
healthy	213.4±45.1	$<$ 0.001	$<$ 0.001

IFCE=isolated focal choroid excavation; SFCT=subfoveal choroidal thickness; P1=isolated FCE groups compared with fellow eye of isolated FCE and healthy groups; P2=fellow eye of isolated FCE compared with healthy groups.
