# Supplementary material for: Risk Factors for Focal Choroidal Excavation Concurrent with Chorioretinal Disease: Evaluated by Spectral-Domain OCT
Source: Ophthalmol Sci. 2024 May 22;4(6):100554. doi: 10.1016/j.xops.2024.100554 (PMC11324813; doi:10.1016/j.xops.2024.100554)
Supplement: Table S5 [file mmc5.pdf]

Table S5. Comparison of SFCT in CFCE, fellow eye of CFCE and healthy groups

	SFCT(μm)	P1	P2
CFCE	293.7±102	-	-
fellow eye of CFCE	246.9±102.5	0.32	-
healthy	213.4±45.1	$<$ 0.001	-

CFCE=complicated focal choroid excavation; SFCT=subfoveal choroidal thickness; P1=complicated FCE groups compared with fellow eye of complicated FCE and healthy groups; P2= fellow eye of complicated FCE compared with healthy groups.
