# Supplementary material for: Risk Factors for Focal Choroidal Excavation Concurrent with Chorioretinal Disease: Evaluated by Spectral-Domain OCT
Source: Ophthalmol Sci. 2024 May 22;4(6):100554. doi: 10.1016/j.xops.2024.100554 (PMC11324813; doi:10.1016/j.xops.2024.100554)
Supplement: Table S6 [file mmc6.pdf]

Table S6. Comparison of SFCT, SECT, width and depth between IFCE and CFCE groups

	IFCE	CFCE	Standard value	P value
SFCT(μm)	333.4±134.8	293.7±102	z=-0.813	P=0.416
SECT (μm)	227.1±112.7	189.1±78.4	z=-1.449	P=0.147
Width(μm)	440.4±264.2	1109.7±906	z=-3.106	P=0.001
Depth(μm)	95.4±78.1	104.4±54.5	t=-0.391	P=0.702

IFCE=isolated focal choroid excavation; CFCE=complicated focal choroid excavation; SFCT=subfoveal choroidal thickness; SECT=sub-excavation choroidal thickness.
