# Supplementary material for: Risk Factors for Focal Choroidal Excavation Concurrent with Chorioretinal Disease: Evaluated by Spectral-Domain OCT
Source: Ophthalmol Sci. 2024 May 22;4(6):100554. doi: 10.1016/j.xops.2024.100554 (PMC11324813; doi:10.1016/j.xops.2024.100554)
Supplement: Table S7 [file mmc7.pdf]

Table S7. Destruction of retina structure in IFCE and CFCE with or without HD.

	With HD	Without HD	Standard value	P value
IFCE/CFCE	2/10	1/14	X^2=5.882	0.023
Mild/severity (destruction of retina structure)	10/6	16/3	X^2=0.023	＜0.001

IFCE=isolated focal choroid excavation; CFCE=complicated focal choroid excavation; SFCT=subfoveal choroidal thickness; HD= Hypertransmission Defects.
