# Supplementary material for: Risk Factors for Focal Choroidal Excavation Concurrent with Chorioretinal Disease: Evaluated by Spectral-Domain OCT
Source: Ophthalmol Sci. 2024 May 22;4(6):100554. doi: 10.1016/j.xops.2024.100554 (PMC11324813; doi:10.1016/j.xops.2024.100554)
Supplement: Table S8 [file mmc8.pdf]

Table S8. Comparison of SFCT, SECT, width and depth between with HD and without HD groups in FCE patients

	With HD	Without HD	Standard value	P value
SFCT(μm)	277.3±108.6	335.8±119.3	z=-1.021	P=0.324
SECT(μm)	151.2±93.2	250.9±75.4	z=-2.931	P=0.003
Width(μm)	1031.2±731.5	608.1±738.1	t=-1.67	P=0.705
Depth(μm)	130±80.2	74.9±40.1	z=-2.03	P=0.043

FCE=focal choroid excavation; SFCT=subfoveal choroidal thickness; SECT=sub-excavation choroidal thickness; HD= Hypertransmission Defects.
